# The sublingual use of atropine in the treatment of clozapine‐induced sialorrhea

**DOI:** 10.1192/j.eurpsy.2021.2061

**Published:** 2021-08-13

**Authors:** H. Becerra Darriba

**Affiliations:** Centro Salud Mental De Tudela - Hospital Reina Sofía, Servicio Navarro de Salud - Osasunbidea., Tudela (Navarra), Spain

**Keywords:** CIS, sublingual atropine, clozapine‐induced sialorrhea

## Abstract

**Introduction:**

Clozapine is a second-generation antipsychotic indicated in treatment-resistant schizophrenia. Patients taking clozapine are likely to experience an increase in salivation or sialorrhea. Clozapine‐induced sialorrhea (CIS) may lead to sleep disturbances or aspiration pneumonia. Treatment options include locally administered anticholinergic medication with atropine ophthalmic drops applied sublingually.

**Objectives:**

To review the current evidence for the effectiveness, safety, side effects and dosage of sublingual application of atropine in reducing or resolving of CIS.

**Methods:**

Systematic review. Data were obtained from PubMed/Medline, EMBASE, PsycINFO, Cochrane Plus, Trip Database, Science Direct and Scopus searches of English-language articles, without restriction for date of publication and study design, reporting the sublingual use of atropine in the treatment of CIS. Large clinical studies with appropriate statistical methods and recruited adults were preferred.

**Results:**

12 selected articles (of 458 references) consisted entirely of case reports and case series. A total of 29 patients with CIS were reported, of whom 24 responded favorably to sublingually administered atropine drops 1% (1-2 drops a day). One limitation of its utilization is the dose-related dry mouth, which can be addressed by lowering the number of drops administered. CIS can occur at different clozapine doses, in various stages of treatment.

**Conclusions:**

The reviewed articles suggest that the use of sublingual atropine is a promising local treatment for CIS. Oral anticholinergic and alpha-2 agonist medications have been used to treat CIS with variable efficacy, but can cause systemic anticholinergic side effects. Further experimental research is needed.

**Disclosure:**

No significant relationships.

